# Lessons from Recent Measles Post-Campaign Coverage Surveys Worldwide

**DOI:** 10.3390/vaccines12111257

**Published:** 2024-11-06

**Authors:** M. Carolina Danovaro-Holliday, Mitsuki Koh, Claudia Steulet, Dale A. Rhoda, Mary Kay Trimner

**Affiliations:** 1 World Health Organization, 1211 Geneva, Switzerland; 2 Biostat Global Consulting, Worthington, OH 43085, USA; dale.rhoda@biostatglobal.com (D.A.R.);

**Keywords:** measles, post-campaign coverage surveys, supplementary immunization activities, coverage

## Abstract

Background: Measles elimination strategies include supplementary immunization activities (SIAs) to rapidly fill immunity gaps. Post-campaign coverage surveys (PCCSs) are recommended to assess SIA coverage. We characterized selected PCCSs performed following recent SIAs, highlighting specific challenges and strengths, and provide recommendations for improvement. Methods: We extracted national SIA data from the global measles/MR SIA database for the period of 2020–2023 and reviewed PCCS reports available at the World Health Organization headquarters. We extracted selected information on PCCS implementation, including information about the implementer, sampling, and main results. Results: Only 15 of 66 countries (23%) with a national-level SIA performed since 2020 had a PCCS report available. We reviewed those reports, plus six more, following three 2019 SIAs with a delayed PCCS and two PCCSs following large subnational SIAs (Kenya 2021 and Yemen 2023). All 24 PCCS reports available were from Gavi-eligible countries, with 15 from South Saharan Africa (Cameroon, the Democratic Republic of the Congo, and Ethiopia had two PCCSs). Eleven (45.8%) PCCSs were conducted within three months of the end of the SIA. All included sampling information and most had percentage of participation. Description of the interviewers’ profiles varied but was limited. PCCS coverage was lower than administrative data in all but two instances. All PCCSs collected data on previous measles vaccination status that would allow exploring indicators on the SIA reaching previously measles zero-dose children. Of the 12 PCCSs reporting coverage among previously measles zero-dose children, nine reported coverage among this group of more than 50% (range: 12% and 91.6%). Conclusion: Even though a PCCS following an SIA is recommended and a requirement in Gavi-supported countries, most SIAs are not followed by a PCCS and, when performed, the timeliness of survey implementation needs improvement. Recent PCCSs were independently conducted and reports included basic survey information, but analysis and presentation of survey results vary particularly for measles zero-dose-related indicators. More guidance and technical support on how to implement PCCSs, including standardization of reports and more in-depth PCCS analyses, may help improve reporting and use of available PCCS data.

## 1. Background

Since the launch of the Expanded Program on Immunization (EPI) 50 years ago, over 154 million vaccine-preventable deaths have been averted, with measles vaccination contributing in a great extent to this achievement [1]. The World Health Organization (WHO) recommends two doses of a measles-containing vaccine (MCV) to be given in routine immunization (RI) [2]. In addition, supplementary immunization activities (SIAs) are a common strategy used to fill immunity gaps resulting from incomplete vaccination through RI services [3,4]. SIAs have been part of measles elimination strategies since their development in the 1990s [4,5]. During an SIA, age- and place-eligible persons, most often children, are vaccinated regardless of their previous vaccination status. In most low- and middle-income countries, SIAs occur every 2–5 years, as the number of measles-susceptible persons accumulate from incomplete vaccination coverage with one or two doses [3,6,7,8]. SIAs have been found to be more equitable in reaching children than routine immunization in several countries [9], and their timeliness and performance have been under much scrutiny recently [10,11]. Most countries have now also introduced rubella vaccination and use measles-rubella-containing (MR) vaccines, with SIAs effectively contributing to rubella control and elimination [12,13]. During the COVID-19 pandemic, not only did MCV1 and MCV2 coverage decline globally [14,15,16], but also many SIAs were cancelled or postponed [17]. MCV coverage is yet to reach pre-pandemic levels globally. As a result, several outbreaks are now occurring across the globe [18,19,20].

To be effective, SIAs need to reach high and homogenous coverage; reaching communities and children previously missed by RI is particularly important. To evaluate the coverage reached by an SIA, the Measles and Rubella Partnership (former Measles Rubella Initiative) recommends implementing an independent post-campaign coverage survey (PCCS) within three months of SIA implementation. For measles and MR SIAs implemented with support from Gavi, the Vaccine Alliance, a PCCS following WHO guidance is required [21,22,23]. A PCCS does not replace intra-campaign monitoring using real-time monitoring of the process, administrative data, and rapid convenience monitoring [3,24,25,26]. However, a PCCS is considered an important tool to estimate the coverage reached by the SIA, to evaluate coverage among children who were previously zero-dose measles vs. those who had been previously vaccinated, and to better understand enablers and barriers to vaccination [22,27].

Concerns have been raised about the quality of vaccination coverage surveys in general and for those following measles campaigns in particular [25,28]. In 2015, the WHO released new survey guidance, first as a working draft and in 2018 as a final product, and accompanying analytical software to overcome some of the challenges [22,28]. Countries have since moved toward conducting independent, probability sampling, vaccination coverage surveys that use probability sampling. This manuscript explores the implementation of PCCSs following measles and MR SIAs since 2020, in addition to selected PCCSs not included in a related publication manuscript published in 2021 [21]. It also characterizes the PCCSs conducted, highlighting specific challenges and strengths, and provides recommendations for improvement.

## 2. Methods

We extracted SIA data from the measles/MR SIA database and reviewed the PCCS reports available at the World Health Organization headquarters as of 11 July 2024. We restricted the analysis to PCCSs conducted since 2020, as a summary of PCCSs conducted between 2017 and 2019 was published by Cutts et al. [21]. However, PCCS reports for 2019 SIAs that were not included in that publication were also included here.

We extracted data on country, vaccine type, SIA date, target age group, estimated target population, administrative coverage, and conduct of the PCCS. For the available PCCS reports, we extracted information on whether it was a national-level survey, dates of field work, sample size, SIA cards or finger marking seen, profile of the implementing partner, sampling approach, length of training, the composition of teams and the supervision provided, and whether the PCCS included questions related to barriers and enablers to vaccination or on reasons for no vaccination. Regarding the results, we extracted information on whether a description of the sample, including the exclusion of certain areas and clusters, was presented. We also extracted MCV RI coverage, SIA overall coverage, and information related to measles zero-dose children, including the proportion receiving their first MCV dose in the SIA, SIA coverage among previously measles zero-dose children, and proportion who remained measles zero-dose after the SIA. Two of the authors (MCD-H and MK) extracted the data in an Excel file, and when one was unsure, both authors reviewed at the report together.

## 3. Results

Review of the WHO SIA database identified 66 countries that had conducted non-outbreak response, national-level, or rolling SIAs between 2020 and 21 June 2024 (Supplemental Table S1). Of them, 15 (23%) had a PCCS with a report available; Ethiopia had two. We also included six additional PCCS reports following 2019 SIAs that were not included by Cutts et al. [21], as well as one PCCS report from Kenya, for a subnational SIA in 2021 and one from Yemen, for a subnational SIA in 2023, but both covered a large proportion of the country. Cameroon and DRC had PCCSs that evaluated 2019 SIAs. In total, our analysis included 24 PCCS reports (Table 1 and Table 2). All PCCSs were implemented at the national level, except for Kenya, which included only the 22 counties where the SIA took place, and Yemen, where the SIA and PCCS were conducted only in the southern governorates. The Somalia PCCS excluded one large northern region, although the SIA was also conducted there. For the Central African Republic, only 57% of enumeration areas available in the list of enumeration areas were deemed accessible and kept in the sampling frame. Eleven PCCSs (45.8%) were implemented within three months of the SIA; four PCCSs (16.7%) were implemented at about one year or more after the SIA. In terms of implementers, four were conducted by the National Bureau of Statistics (NBS) and all but two at least mentioned collaborating or using a sampling frame from the NBS. Twenty-three PCCSs reported having performed a weighted analysis, although in one it was unclear if weighting was performed according to the sampling strategy or only to aggregate the country-level estimates. For Syria, weighting was not performed as per NBS guidance due to the current situation of moving populations and unavailability of accurate population data at any administrative level. Ten (43%) were led by independent consultants or organizations and four (20.8%) were led by an academic institution or a research organization.

Twenty-one (87.5%) reports indicated having referred to the 2018 WHO Vaccination Coverage Cluster Survey Reference Manual, with one referring to an outdated version (2005), and most described the key sampling elements. All but two reports described exclusions or furnished the response rate. Sample sizes varied, but most PCCSs were large, with all having >1000 children. Regarding field implementation, dates were explicitly stated in all but one report. The description of the composition of the teams varied broadly, with the profile and gender of interviewers rarely described (data not displayed in the table, as the reports varied widely). Seventeen PCCS reports mentioned ethical clearance being obtained and two (Chad and Kenya) indicated that the survey was considered a programmatic evaluation and thus ethical clearance not required. The other five reports had no mention of ethical review, although explicit information on informed consent was available for three (the two PCCSs from Cameron and the one in Nigeria) of these five. Copies of the questionnaires were included in 19 reports (79.2%). Of relevance, all but one of the PCCSs were performed using computer-assisted personal interviewing (CAPI) and at least seventeen (70.8%) collected GPS coordinates. We did not explicitly extract information on whether geospatial analyses were conducted, but from reading the reports, we noted that maps were not frequently included. Finally, all PCCSs included a section on reasons for no vaccination, with two mentioning the Behavioral and Social Drivers of Vaccination (BeSD) framework.

The main PCCS results and comparisons to administrative data and routine coverage with the first dose of a measles-containing vaccine (MCV1) are included in Table 2. PCCS SIA coverage was lower than the administrative coverage included in the SIA database, apart from DPR Korea and the accessible areas of Syria. Regarding ascertainment of SIA vaccination, 19 had vaccination cards or finger marking as proof of vaccination in the SIA (range: 6.9–81.1%). One third of the PCCSs reported SIA coverage that was below the WHO/UNICEF Estimate of National Immunization Coverage (WUENIC) for one dose of a measles-containing vaccine. Twenty PCCS reports (83.3%) included at least one of the indicators related to zero-dose measles. Of the 12 PCCSs reporting coverage among previously measles zero-dose children, nine had coverage of more than 50% (range: 12% and 91.6%). DPR Korea and Nepal explored this indicator but unvaccinated children in the sample were extremely few. Only the Zambia PCCS report did not allow ascertaining whether the collected data would allow calculating at least one of the three measles zero-dose SIA coverage indicators included in our table.

Only one anonymized database was available to WHO-HQ. This PCCS had been re-analyzed, in 2023, using the tool Vaccination Coverage Quality Indicators (VCQI) [29] upon request from stakeholders. In this PCCS, which excluded one large northern region and security-compromised areas, SIA coverage among children previously vaccinated with either one or 2+ measles doses was strikingly higher than among those who had no previous MCV dose. Table 3 illustrates the VCQI output for a given age-group.

Secondary analyses were conducted to better understand SIA coverage heterogeneity. Table 4 describes measles SIA coverage, the number of children in the sample, and the number of clusters, by state. It then indicates the number of clusters where all surveyed children were found vaccinated, those where the proportion was >50% but <100%, and then the clusters where the proportion of vaccinated children was ≤50%. The design effect (DEFF) and intra-cluster correlation coefficient (ICC) are also included for each stratum. Both parameters were high, with the DEFF ranging from 10.8 to 28.5, which is largely above the parameters used in sample size calculations. The large value observed here is due to the high ICC and an unusually high number of respondents per cluster.

**Figure 1:**
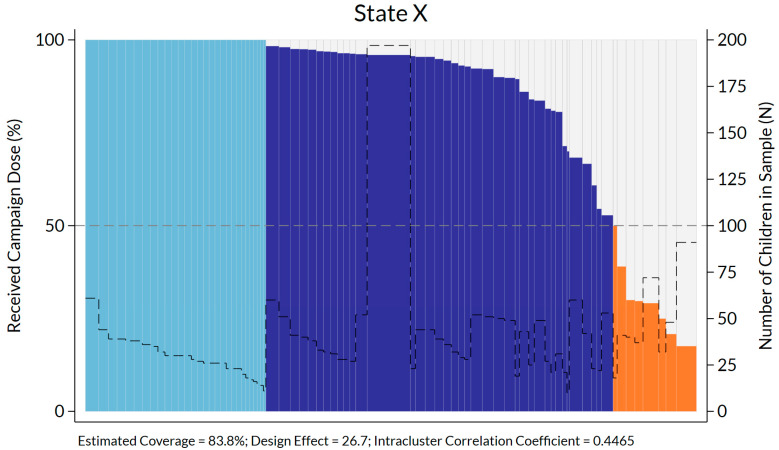
Organ pipe plot of proportion of children vaccinated per cluster in State X. Somalia (minus one large northern region one large northern region) PCCS 2023.

An organ pipe plot [21,30], as recommended by the WHO Vaccination Coverage Survey Manual, was produced for all 6 states included in the PCCS (Figure 1—illustrative). Cluster-level SIA proportion vaccinated (indicated by the individual columns) varied considerably across State X.

Figure footnote: Clusters are sorted left-to-right in descending order of coverage. In this representation, clusters with 100% sample coverage are light blue, those with 50.01–99.99% are dark blue, and those with 0–50% are shown in orange. The thin gray dashed line that varies in height from cluster to cluster indicates the number of respondents per cluster and its values are read using the right vertical axis.

Only two other PCCS reports reviewed for this work included calculation of the intra-cluster correlation coefficient (ICC) or design effect (DEFF), parameters that are useful to inform sample size calculations in future PCCS.

## 4. Discussion

Though measles SIAs are performed in many parts of the world, few countries use PCCSs to evaluate their performance. All PCCSs for which the WHO had access to reports were conducted in Gavi-eligible countries, and most in Sub Saharan Africa. If we compare our findings with previous reviews [21,25], the use of probability sampling and weighted analysis is encouraging; most PCCSs are explicit in indicating that current WHO guidance is being considered. The independence of implementers and quality of reports has also improved compared to previous studies.

Many challenges remain, however. Not all countries can implement a PCCS, and this is evident from the fact that only Gavi eligible countries had PCCS reports available. Also, the timing of PCCS implementation is of particular concern. Only about half of the PCCSs explored here were implemented within three months of the SIA, though our findings are confounded by the limitations to field activities imposed by the COVID-19 pandemic.

Administrative coverage levels tend to overestimate campaign coverage compared to coverage obtained from PCCS. This finding is not surprising. Administrative coverage can be affected by inaccurate denominators and errors in tallying and aggregating doses. For SIAs, vaccination of children outside the target age while tallying them as within the age range is not uncommon.

Only five PCCS coverage levels were above 90%, and only one >95%. We also observed that measles SIA coverage was lower than routine MCV1 coverage for a third of the PCCSs included. This was unexpected, as SIAs, by their nature, conduct intensive outreach and social mobilization. This warrants more exploration, as it may also be that as documentation of vaccine doses received improves, children who are already vaccinated may be less likely to go for an additional dose.

The next questions after seeing coverage that is insufficient to stop measles transmission are: where coverage was not reached and why. While PCCSs can provide clues, these answers would be better addressed by better intra-campaign monitoring and rapid convenience monitoring before stopping vaccination. We did not explore these aspects of SIA implementation but would argue that they need strengthening judging by the SIA coverage reached in most recent campaigns and their heterogeneity in reaching previously unvaccinated children [4,21,27,31]. Furthermore, to our knowledge, only campaign coverage is broadly accepted and recommended as an indicator of measles SIA quality and impact. The establishment of more criteria to assess SIAs was recommended by the Measles & Rubella Strategic Framework 2021–2030 published at the beginning of the decade [32].

Conducting a PCCS is not always easy. The PCCSs included here tend to be large, with over 1000 children. The more clusters in a survey, the more resources are needed to reach the different areas. In addition to the challenges linked to the normal work of reaching selected places, interviewing families takes time and effort. Four reports explicitly mentioned the violence that survey teams faced, with one survey team even being kidnapped in a country in central Africa. To this end, one should consider under what situations a PCCS may not be the best use of resources or may not warrant the risk that field work may entail. Also, complementary monitoring tools may need to be used in insecure areas and others that are not included in normal sampling frames. Decision support tools may be worth considering. Time elapsed between the SIA and the PCCS may be a consideration. Senegal implemented a selective SIA in 2021. It was an SIA of particular interest because it involved screening and listing children aged 9 to 59 months (performed in Oct 2021) and then vaccination of these children at fixed and outreach sites (in November 2021); children not on the lists but who came to a site without documentation of having previously received two doses of MR vaccine were also to be vaccinated. Unfortunately, a PCCS was not conducted until 2023, and at the time of this writing, in July 2024, the report is not available. Another example where a PCCS may not be the best option is when an SIA is known to not have reached high coverage, using administrative data and a narrative of the issues, complemented or not with rapid convenience monitoring, may be a better option. This was the case in Indonesia outside of Java in 2017–2018 and the Gambia in 2022. Another example might be that of South Sudan, where after performing an SIA in 2023 decided to prioritize campaign evaluation and rapid field assessments, and their PCCS was paired with a planned RI vaccination coverage survey that was scheduled for late 2023, after the rainy season. This survey is yet to be implemented. Recent outbreaks affecting mainly children aged 1–4 years suggest that coverage was likely insufficient [33]. More needs to be done to triangulate data from intra-campaign monitoring and administrative coverage with PCCS findings to better inform the proposed decision support algorithms [3,31].

When PCCSs are implemented, exploiting the findings and the available data should be of utmost priority. The WHO and partners make available analytical software and a detailed list of indicators [29]. However, most of the surveys described in the reports accessible to us did not use them. This may reflect limited awareness or other barriers that we need to further explore. In addition to coverage and the availability of sociodemographic variables that would allow exploring factors associated with no vaccination, most PCCSs now collect GPS coordinates. They also have information about reasons for no vaccination, and more recent PCCSs are starting to use the Behavioral and Social Drivers of Immunization (BeSD) [34] framework to explore barriers and enablers to vaccination.

Some of the countries included here used survey data to conduct exploratory analysis on factors linked to not being vaccinated. Nigeria explored geographic differences in measles vaccination in RI vs. SIAs [27]. The analysis showed that the SIA reached more homogenous coverage than the RI, but that there are areas where many children are missed by both approaches. Furthermore, geolocated campaign and RI coverage data can be extremely useful to modelers when modeling disease transmission and outbreak risk, which can help to inform disease control targeting [10,11,31,35,36,37,38,39]. However, unlike well-established household survey programs, there is no PCCS database repository. The example presented here allowed exploring heterogeneity in coverage. The results, which represent areas that were accessible to surveyors, warrant better exploring why coverage among measles zero-dose children was so much lower than that for those who had been previously vaccinated. Countries should be encouraged to make anonymized databases available to the WHO, the IA2030 Measles and Rubella Partnership, and other stakeholders, as is done by most household surveys. Furthermore, we support the initiative by the IA2030 Partnership to establish data analysis cooperation in countries conducting coverage surveys with additional analyses performed by local analysts, close in time to the PCCS, and engaging with EPI programs to promote data use.

Our analysis has several limitations. First, we included only national SIAs that were not for outbreak response. We may have accidentally left out SIAs that should have been included. Second, we may not have the reports of all PCCSs conducted. This is less likely for Gavi-eligible countries, as the authors reached out to Gavi and the US Centers for Disease Control and Prevention (CDC) to seek reports. However, non-Gavi eligible countries may not share PCCS reports with the WHO. Third, we selected only a few characteristics of the PCCS and the reports from a long list of recommended items in the survey checklist recommended by Cutts et al. [21] and adopted by Gavi. Also, we did not compare the reports to the elements recommended in the recently introduced “Preferred Reporting Items for Complex Sample Survey Analysis” (PRICSSA) checklist [40]. Fourth, two authors extracted data from the PCCS reports, and as they were not structured according to our list of items, we may have misrepresented some data; we did not attempt to reach PCCS teams for clarifications. Lastly, having an item in a report does not guarantee good survey implementation. There are many challenges to conducting field enumeration and sampling, identifying pre-selected clusters boundaries and pre-selected households, and to interviewing people, particularly to ascertain vaccination status when a card or other documentation is not available. Caregivers’ ability to accurately recall vaccination experience for each of their children can be challenging, and more so the longer the time lag between the SIA and the survey. Analysis of data collected using complex sampling, particularly the appropriate weight calculations, and use of sample design parameters in statistical packages is challenging. We did not have access to any analytical codes, except for the VCQI code used for one PCCS performed by two co-authors (DR and MKT). Nevertheless, we believe that our main findings remain solid: not all SIAs are followed by a PCCS and, when performed, timeliness remains a concern; more PCCSs appear to be following current recommendations; survey coverage tends to be lower than administrative SIA results; and more analysis could be performed with the data that are collected by PCCS analysts and, if made available, by measles modelers and other stakeholders.

Gavi contracted out two PCCSs in 2024, one for Burkina Faso and one for Laos, in an attempt to ensure PCCS timeliness and quality survey implementation. Neither report is available for review yet. This work will present an opportunity to explore the pros and cons of a centralized contracting approach, as well as to explicitly triangulate PCCS results with other data to better inform decision making.

The WHO is coordinating a thorough update to the 2018 Vaccination Coverage Cluster Survey Reference Manual [22] and accompanying software tools [29] in 2024–2026. This presents an opportunity to further engage with countries and the IA2030 Measles and Rubella Partnership to ensure that the revised guidance includes options for areas where regular sampling is problematic, clearer standardization for reports, more guidance on analyses beyond descriptive statistics, and more hands-on capacity-building activities if a stakeholder analysis identifies this as useful.

Measles is so exceedingly contagious that very high vaccination coverage is required to prevent outbreaks from spreading through a population. High-quality, independent, probability-based assessments of campaign coverage will be useful both for refining SIA planning and execution to consistently achieve high coverage rates, especially among measles zero-dose children.

## Figures and Tables

**Table 1:** Summary of basic characteristics of post-campaign coverage surveys (PCCSs) following measles supplementary immunization activities.

				Measles Supplementary Immunization Activities (SIAs)				Post-Campaign Coverage Surveys (PCCSs)															
				Basics				Basics					Sampling										
#	Year of PCCS	Country	WHO Region	Target Age Group in Months	Estimated Target Population	Admin Coverage %	SIA Month/Year	Month/Year of Field Work	Delay Between SIA & PCCS (in Months)	Lead Implementer	Ethical Clearance Mentioned	Questionnaire/Data Collection Form Included in Report	# of Clusters Selected/Included	# of Households Interviewed	# of Children Included	Description of Response Rates of Clusters	Description of Response Rates of Households	Primary Sampling Units	Household Enumeration Done	Involvement of NBS/CSO	Data Collection Tools	GPS Coordinates Collected	Software Used for Analysis
1	2019	Democratic People’s Republic of Korea	SEAR	9 months-15 years old	5,873,914	99.7	Oct-19	Winter	NA	Country-led	Yes	Yes	41	1312 (31 HH in each cluster)	1180	Yes	Yes	Census enumeration blocks	Yes	Yes	CSPro	No	SPSS
2	2019	Zimbabwe	AFR	9–59 months	1,776,546	91	Sep-19	Nov-19	2 months	Not specified	Yes	Yes	470	8110 planned *actual number included missing	3796 planned *actual number included missing	No	No	Enumeration areas	Yes	Yes	ODK	NR	Stata
3	2020	Burkina Faso	AFR	9–59 months	3,078,334	106	Nov-19	Aug–Sept 2020	9–10 months	Independent consultant	Yes	No	548	17,650	8457	Yes	Yes	Enumeration areas	Not mentioned	Sampling frame from NBS	ODK	NA	SPSS and Stata
4	2020	Cameroon	AFR	9–59 months	3,339,090	92	Dec-19	June–July 2020	6–7 months	Research Organization	Not mentioned. Informed consent was obtained.	Yes	396	4866	4671	Yes	Yes	Enumeration areas	Yes	Sampling frame from NBS	ODK	Yes	SPSS
5	2020	Ethiopia	AFR	9–59 months	14,181,143	102.8	June–July 2020	Oct 2020–Jan 2021	4–6 months	Research Organization	Yes	Yes	1016 Tigray was excluded	10,143	12,867	Yes	Yes	Enumeration areas	Yes	Sampling frame from NBS	CSPro	Yes	Stata, VCQI
6	2020	Uganda	AFR	9 months to less than 15 years old	18,200,970	107	Oct-19	Dec 2019–Jan 2020	2–3 months	Academic Institution	Yes	No	363	5174	12,025	Yes (written as coverage rate)	Yes	Enumeration areas	Yes	Sampling frame from NBS	ODK	NA	Stata
7	2021	Central African Republic †	AFR	6–59 months 5–10 years	1,209,256	102 for 6–59 m 85.5 for 5–10 yrs	Phase 1: Mar-2020 Phase 2: Aug-2020	Dec 2020 Bangui, March–April 2021 rest	8–11 months	Independent consultant	Yes	Yes	240	3172	5674	Yes	Yes	Enumeration areas	Yes	Sampling frame from NBS	ODK	Yes (difficulties noted)	SPSS
8	2021	Chad	AFR	9–59 months	1,792,830 (Phase1) 1,623,518 (Phase2)	108.8 (Phase1) 107.5 (Phase2)	Jan- 2021 (Phase1) Mar-2021 (Phase2)	Mar–April 2022	~1 year	Independent consultant	The survey technical committe considered that the survey was not reasearch and thus it was not needed to present the protocol to an ethical comittee. Informed consent was obtained.	Yes	695	15,793	11,601	Yes	Yes	Enumeration areas, with segmentation	Yes	Yes	ODK	Yes, at least for clusters	SPSS and Stata
9	2021	Democratic Republic of the Congo	AFR	6–59 months	18,167,926	101.6	Oct-Dec 2019	Nov–Dec 2021	~2 years	Independent consultant	Yes	Yes	728	81,740	21,575	Yes	Yes	Enumeration areas	Yes	Yes	CSPro	Yes	SPSS, EpIInfo
10	2021	Kenya ‡	AFR	9–59 months	3,374,464	111	June–July 2021	Jul-21	<3 months	NBS	Considered programmatic evaluation. No ethical clearance was sought. Informed consent was obtained.	No	477	4404	5409	Yes, but by Region	Yes	Enumeration areas	Not for the PCCS. Sample was from available up-to-date sampling frame maintained by NBS.	Yes	Survey Solution application with digitization of the questionnaire and integration of maps.	Yes	Not mentioned
11	2021	Nepal	SEAR	9–59 months	2,548,336	101	Feb–Mar 2020 (Phase 1) Mar–July 2020 (Phase 2)	Sept–Nov 2021	>1 year	Family Welfare Division Department of Health Services MOH	Yes	Yes	342	7180	3715	Yes	Yes	List of wards (some were merged or segmented)	Yes	Yes	CSPro	Yes	SPSS
12	2021	Pakistan	EMR	9 months-15 years old	90,262,980	105	Nov-21	Nov 2021–Jan 2022	<3 months	Contech International Health Consultants	Yes	Yes	1584	15,840	31,871	Yes	Yes	Enumeration areas	Yes	Yes. Technical and sampling frame	Proprietary android-based CAPI application from implementing partner	No	Stata, VCQI
13	2021	Zambia	AFR	9–59 months	3,398,230	91.3	Nov-20	Oct-21	>1 year	Not specified	No	No	272	5155	4590	No	Yes	Enumeration areas	Yes	Yes	The questionnaire was programmed into a CAPI application using survey solutions, a World Bank software application		Not mentioned?
14	2022	Burundi	AFR	9–59 months	1,683,300	93	Jan-22	Oct–Nov 2022	10–11 months	NBS	Not mentioned	Yes	540	32,177	20,618	Yes	Yes	Ennumeration areas	Yes	Yes	KoboCollect	Yes	SPSS and Stata
15	2022	Madagascar	AFR	6–59 months	4,355,433	95.11	May–June 2022	Aug–Sept 2022	3 months	International consultant with Directorate of Demography and Social Statistics (DDSS) of the NBS	Yes	Yes	123	3055	1421	Yes	Yes	Enumeration areas	Yes	Yes	CSPro	Yes	SPSS and Stata
16	2022	Somalia	EMR	children of 0–59 months for tOPV, 06–59 months for MCV and Vitamin A and 12–59 months for deworming	2,566,955	90	Nov-22	Feb-23	3 months	Independent consultant/cabinet	Yes	Yes	450	17,539	21,740	No	No	Lists of accessible areas (not mentioned in detail)	Not mentioned	Not mentioned	Survey123	Yes	SPSS, Stata, ArcGIS
17	2022 or 2023	Syrian Arab Republic	EMR	6 months-5 years	2,494,498	75.62	Oct–Nov 2022	Dates not provided. 2023?	NA	Independent consultant	Yes	No	99	5982	3581	Yes	Yes	Sub-districts	Yes	Yes, technical advise	Paper-based	No	EpiInfo
18	2023	Cameroon	AFR	9–59 months	5,564,940	94.38	Jul-23	Sept–Oct 2023	<3 months	NBS	No. Informed consent was obtained.	Yes	395	5711	3546	Yes	Yes	Enumeration areas	Yes	Yes	CSPro	Yes	SPSS, Stata, VCQI
19	2023	Democratic Republic of the Congo	AFR	6–59 months	6,454,490 (May) 7,359,339 (August) 5,273,383 (September)	103.6 (May) 85.7 (August) 101.1 (September)	Three phases: April, June and Aug 2023.	October–December 2023 in Block 1, Phase 2 in Block 2 and part of Block 3 from January to March 2024, then Phase 3 in the rest of the provinces in March–April 2024.	6 to 10 months	Independent consultant	Yes	Yes	728	10,920	9627	Yes	Yes	Enumeration areas	Yes	Yes	CSPro	Yes	SPSS, EpIInfo
20	2023	Malawi	AFR	9–59 months	3,169,522	82.4	May-23	Jun-23	<3 months	International consultant and NBS	Yes	Yes	205	4715	8485	Yes	Yes	Enumeration areas	Yes	Yes	CSPro	Yes	Stata, VCQI
21	2023	Niger	AFR	6–59 m or 9–59 m depending on district	5,098,682	105	Dec 2022–Jan 2023	Mar–April 2023	3 months	Independent consultant	Yes	Yes	270	5334	4655	Yes	Yes	Ennumeration areas	Yes	Yes	ODK	Yes	SPSS
22	2023	Nigeria	AFR	9–59 months	4,298,149 (October) 1,090,330 (November) 5,021,611 (December)	96.67 (October) 103.95 (November) 4,823,266 (December)	Oct 2023–Jan 2024	Dec 2023–Jan 2024	<3 months	NBS	No. Informed consent was obtained.	Yes	740 (560 in 14 states plus 180 in 6 local area governments in Borno)	7399	6987	Yes	Yes	Enumeration areas	Yes	Yes	CSPro	Yes	Stata, VCQI
23	2023	Yemen ‡	EMR	6–59 months	1,267,083	91	September–October 2023	Oct-23	<3 months	Research Organization	Yes	Yes	1547	18,564	29,549	Yes	Yes	Harahs	No	No	ODK	No	Excel
24	2024	Ethiopia	AFR			98.7	Dec 2022–April 2023	Dec 2023–Jan 2024	>1 year	Research Organization	Yes	Yes	1272	12,702	15,763	Yes	Yes	Enumeration areas	Yes	Sampling frame from NBS	Kobo Toolbox	Yes	Stata, VCQI

AFR = World Health Organization African Region; EMR = World Health Organization Middle Eastern Region; SEAR = World Health Organization South-East Asian Region NBS = National Bureau of Statistics; NA = not available; NR = not reported, but potentially available; VCQI = Vaccination Coverage Quality Indicators tool; † Areas considered secure; ‡ Subnational.

**Table 2:** Summary of main results of post-campaign coverage surveys (PCCSs) following measles supplementary immunization activities (SIAs) and related coverage levels.

						Results						
#	Year of PCCS	Country	WHO Region	MCV1 Coverage in Year of Start of SIA	Admin Coverage %	SIA Cards or Finger Marking Seen	SIA Coverage (95% CI)	% Routine Immunization (RI) Cards Seen (Age Group If Not All)	RI MCV1 Coverage % (95% CI) (Age Group If Not All)	SIA Dose Was the First MCV Dose Received by Child % (95%CI)	SIA Coverage Among Zero-Dose % (95%CI)	Child Remained Measles Zero-Dose After SIA % (95%CI)
1	2019	Democratic People’s Republic of Korea	SEAR	98	99.7	Almost all were verified by record in Primary Health Facility. Only recall were 7 children who had records in a different health facility	99.9% (99.09–99.87)	NA	81% yes, 18.6% unsure, 0.4% no	NR	NA	NA
2	2019	Zimbabwe	AFR	85	91	NR	78.7% (77.35– 79.98)	NA	NA	NR	NR	NR
3	2020	Burkina Faso	AFR	88	106	38.7% (87.8% received one)	84.4% (83.6–85.2%)	93% 12–23 months 81% 24–35 months	87,8% 12–23 m 84.8% 24–35 m	NR	NR	4.80%
4	2020	Cameroon	AFR	61	92	8.30%	69.7% (68.34–71.01)	NA	NR	35.70%	NR	NR
5	2020	Ethiopia	AFR	59	102.8	81.10%	81.5% (80.0–83.0%)	NA	80.30%	NR	56.70%	8.10%
6	2020	Uganda	AFR	87	107	48.5 (78.4% received one)	94.4% (93.0–95.5)	25.30%	85.40%	12.00%	NR	2.60%
7	2021	Central African Republic †	AFR	41	102 for 6–59 m 85.5 for 5–10 yrs	25% (83% received one)	94.6% (92.9–96.0%)	NA	25.2% 12–23 m 15.3% 24–35 m	NR	NR	3.40%
8	2021	Chad	AFR	53	108.8 (Phase1) 107.5 (Phase2)	11% (19% reported not having received a card)	77.4% (74.8–79.8%)	NA	Available as an analysis added later, not in main report. 41.1% 12–23 m 28.2% 24–35 m	NR	73.5% (IC95%:70.2–76.5%)	20% (IC95%: 17.6–22.5%)
9	2021	Democratic Republic of the Congo	AFR	65	101.6	6.89%; 4.93% (correctly filled in)	87.52% (87.50–87.53%)	NR	80.70%	NR	61.06%.	7.61%
10	2021	Kenya ‡	AFR	90	111	NR	84.20%	NA	NA	NR	80.10%	NR
11	2021	Nepal	SEAR	87	101	43%	84% (82–87) Phase 1: 87% (85–89) phase 2: 81% (77–85)	51.40%	95.50%	Small sample size of previously zero-dose	Small sample size of previously zero-dose	NR
12	2021	Pakistan	EMR	81	105	80%	93.6% (92.7–94.4)	47%	71.5% (and 10% unkown status) 79% for children under 2 years	NR	NR	NR
13	2021	Zambia	AFR	96	91.3	NA	68.90%	63% had documented evidence	88.5	NA	NA	NA
14	2022	Burundi	AFR	89	93	15%	88.60%	91.8% 12–23 m 86.1% 24–35 m	85.90%	NR	NR	NR
15	2022	Madagascar	AFR	44	95.11	56.30%	65.3% (56.8–72.9)	NA	69.50%	NR	NR	19.20%
16	2022	Somalia	EMR	46	90	NA	86.0 (83.7–88.0)	25% 12–23	65%	NR	33.1% 14.1% 12–23 m 37.2% 24–35 m	NR
17	2022 or 2023	Syrian Arab Republic	EMR	52	75.62	54.40%	80.7% (79.35%–81.94%)	NA	82.8 12–23 m 93.6 24–59 m	NR	54.30%	NR
18	2023	Cameroon	AFR	71	94.38	31.8% (84.9% received a card)	69.5 (66.4–72.5)	NA	NR	22.10%	62.30%	13.3%
19	2023	Democratic Republic of the Congo	AFR	52	103.6 (May) 85.7 (August) 101.1 (September)	12.88%	94.60% (94.60%–94.61%)	~3.5% (3.47% vaccinated by card seen)	78.30%	NR	75.01%	NA
20	2023	Malawi	AFR	87	82.4	38.8%	75.7% (72.3–79.0)	NA	NR	NR	27.6% for 12–35 m	NR
21	2023	Niger	AFR	65	105	71.8% (86.2% received one)	92.7% (90.8–94.1)	NA	NA	NR	91.60%	4.60%
22	2023	Nigeria	AFR	60	96.67 (October) 103.95 (November) 4,823,266 (December)	47% card and 9.9% finger marking	87% (84–90)	NR	NR	12%	NR	NR
23	2023	Yemen ‡	EMR	45	91	NA	84.2% (95% CI: 83.8–84.6)	NA	NR	NR	12%	NR
24	2024	Ethiopia	AFR	55	98.7	16%	87.10%	3940 (~25%)	73.80%	14.00%	72%	NR

AFR = World Health Organization African Region; EMR = World Health Organization Middle Eastern Region; SEAR = World Health Organization South-East Asian Region; NBS = National Bureau of Statistics; NA = not available; NR = not reported, but potentially available; † Areas considered secure; ‡ Subnational.

**Table 3:** Measles SIA vaccination coverage by previous vaccination status, children aged 24–35 months, Somalia (minus one large northern region) PCCS 2023.

Number of Measles Vaccine Doses Prior to SIA	Vaccinated During SIA (%)	95% CI (%)	Vaccinated During SIA (Weighted N)	Weighted N
Zero	37.2	(31.8, 42.8)	81	217
1 Dose	92.8	(89.5, 95.1)	540	582
2+ Doses	90.9	(86.6, 94.0)	812	894
Somalia 2023 (Total)	84.7	(81.6, 87.3)	1433	1692

**Table 4:** Measles SIA coverage, the number of children in the sample, and the number of clusters, by state. Somalia (minus one large northern region) PCCS 2023.

State	% Vac’d During SIA	Total # Children	Total # of Clusters	# Clusters with 100% Children vac’d’	# Clusters with >50–99.9% Children vac’d	# Clusters with ≤50% Children vac’d	DEFF	ICC
Banadir	89.8	3604	74	13	57	4	11.7	0.2216
Galmudug	86.1	3338	76	10	61	5	10.8	0.1634
Hirshabelle	82.6	4372	76	17	47	12	28.5	0.4284
Jubbaland	83.8	2781	76	29	39	8	26.7	0.4465
Puntland	88.6	3279	73	18	52	3	14.0	0.2361
Southwest	87	3542	75	20	48	7	17.7	0.2791
Total	87	20,916	450	107	304	39	21.3	0.3116

DEFF: design effect; ICC: intra-cluster correlation coefficient. For PCCS, ICC values of 1/6 = 0.167 are considered conservative [21].

## Data Availability

Data is contained within the article.

## Supplementary Materials

The following supporting information can be downloaded at: https://www.mdpi.com/article/10.3390/vaccines12111257/s1. Table S1: List of measles campaigns, global measles supplemental immunization activity (SIA) database.
